# Atophan, a New Physiologically and Clinically Approved Drug in Gout and Articular Rheumatism

**Published:** 1911-07

**Authors:** 


### Atophan, a New Physiologically and Clinically Approved Drug in Gout and Articular Rheumatism

Professor W. Weintraud of the Municipal Hospital at Wiesbaden, Germany, an internationally famed resort for the treatment of gout, reports in *"Therapie der Gegenwart,"* March, 1911, the results of his experimentation with Atophan. Under this name the 2-phenyl-chinolin-4-carbolic acid, which Nicolaier and Dohrn found to exercise a specific and powerful influence on the uric acid metabolism (*"Deutsches Archiv f. klinische Medizin,"* 1908, Vol. 93.) has recently been introduced into therapeutics by the Schering Chemical Works of Berlin-Weintraud, at the hand of a great number of metabolic data, confirms in every way the findings of Nicolaier and Dohrn in healthy individuals, and after employing Atophan for nearly two years in numerous cases of gout, has come to the conclusion that this drug eliminates uric acid from the system in a manner and degree never before equaled by any other therapeutic agent. This action is observed both under purin-containing and purin-free diet. He finds Atophan primarily indicated in the acute attack of gout, in which it promptly relieves the pains and causes speedy retrogression of the inflammatory symptoms and swellings, being not only the equal, but in reliability, promptness and absence of by-effects, the superior of colchicum preparations. Weintraud also advocates the periodical giving of Atophan in chronic gout as reducing the deposits and materially relieving the inflammatory symptoms.

Tachernikow and Magat of the Hospital Clinic University of Charkow, Russia (*Charkower Medizinisches Journal,* 1910) and Heller of the Hasenheide Hospital, Berlin (*Berliner Klinisches Wochenschrift,* 1911, No. 12) clinically confirm these findings and have had excellent results with Atophan also in non-uratic joint development, especially in acute articular rheumatism. Heller considers Atophan a more valuable antirheumatic than acetylsalicylic acid and finds it free from the disagreeable by-effects (constipation, and the like) of the latter. In fact, Atophan has a favorable influence upon the stool of the patients.

